# Development and Application of a Dual-Readout RPA-PfAgo System for Rapid Detection of *Streptococcus agalactiae* in Bovine Milk

**DOI:** 10.3390/vetsci13060561

**Published:** 2026-06-06

**Authors:** Xujie Zhao, Yuying Du, Qianlei Zhu, Yang Cai, Lin Chen, Jingjing Li, Mingzhu Zhou, Bingze Jiao, Yilin Bai, Lei Wang, Yanwei Li, Mingcheng Liu, Jianhe Hu, Ke Ding, Xiaojing Xia

**Affiliations:** 1 College of Animal Science and Veterinary Medicine, Henan Institute of Science and Technology, Xinxiang 453003, China; 2 Ministry of Education Key Laboratory for Animal Pathogens and Biosafety, Zhengzhou 450002, China; 3 Agricultural Comprehensive Administrative Law Enforcement Brigade of Jinpu New Area, Dalian 116200, China; 4 Laboratory of Indigenous Cattle Germplasm Innovation, School of Agricultural Sciences, Zhengzhou University, Zhengzhou 450001, China

**Keywords:** *Streptococcus agalactiae*, recombinase polymerase amplification (RPA), PfAgo, fluorescence detection, lateral flow dipstick, molecular detection

## Abstract

*Streptococcus agalactiae* is a major contagious pathogen of bovine mastitis and causes substantial economic losses in the dairy industry. In this study, a dual-readout RPA-PfAgo detection platform targeting the conserved *cfb* gene of *S. agalactiae* was established and optimized. Seven pairs of RPA primers were designed and screened to construct the Basic-RPA assay, and six guide DNAs (gDNAs) together with a specific probe were evaluated for PfAgo-assisted detection. Field validation was performed using 153 bovine milk samples collected from five dairy-farming regions in China, and assay performance was compared with bacteriological culture and a standardized quantitative PCR (qPCR) assay. The Basic-RPA assay achieved optimal amplification at 37 °C for 30 min, with a detection limit of 1 × 10^−3^ ng/µL and no cross-reactivity with non-target bacteria. The optimized RPA-PfAgo-RTF assay detected as few as 10 copies/µL, whereas the RPA-PfAgo-LFD assay detected 100 copies/µL, and both formats showed high analytical specificity. In field milk samples, bacteriological culture detected 21 positive samples, whereas standardized real-time PCR (qPCR), RPA-PfAgo-RTF, and RPA-PfAgo-LFD each detected 33 positive samples. When compared with bacteriological culture as a conventional comparator, all three molecular assays showed 100.00% positive agreement, 90.91% negative agreement, and a Kappa value of 0.733. In addition, RPA-PfAgo-RTF and RPA-PfAgo-LFD were completely concordant with the standardized qPCR assay across all 153 samples. These results indicate that the dual-readout RPA-PfAgo platform is a rapid and reliable molecular tool for detection of *S. agalactiae* in bovine milk.

## Simple Summary

*Streptococcus agalactiae* is an important contagious pathogen of bovine mastitis and can cause major economic losses in dairy herds. Rapid detection is essential for timely diagnosis and control, but conventional culture methods are time-consuming and may miss low-level infections. In this study, we developed a dual-readout detection platform that combines recombinase polymerase amplification (RPA) with *Pyrococcus furiosus* Argonaute (PfAgo) and provides two result formats: real-time fluorescence (RPA-PfAgo-RTF) and lateral flow dipstick (RPA-PfAgo-LFD). Both assays showed high specificity for *S. agalactiae*, with detection limits of 10 copies/µL and 100 copies/µL, respectively. When evaluated using 153 bovine milk samples from five dairy-farming regions in China, both assays were completely concordant with a quantitative PCR method and yielded more molecular-positive results than bacteriological culture. These findings indicate that the RPA-PfAgo platform is a rapid and practical molecular tool for detecting *S. agalactiae* in bovine milk, with the fluorescence format being more suitable for laboratory testing and the lateral flow format offering advantages for field screening.

## 1. Introduction

*Streptococcus agalactiae* (*S. agalactiae*), also known as Group B Streptococcus (GBS), is a Gram-positive bacterium commonly found in humans and animals. It serves as a significant causative agent of mastitis in dairy cattle and is also a primary pathogen responsible for neonatal sepsis, meningitis, and puerperal infections in pregnant women [1]. In the dairy farming industry, mastitis caused by *S. agalactiae* not only reduces milk production and compromises the quality of dairy products, but also increases the somatic cell count in milk, which significantly impairs the processing characteristics and economic value of dairy products [2,3,4]. Due to the asymptomatic or subclinical nature of the early stage of infection, conventional clinical practices often fail to detect such cases in a timely manner. This delay in identification facilitates the transmission of the pathogen within the population, thereby exacerbating economic burdens and heightening public health risks. Therefore, the establishment of a rapid, highly sensitive, and specific detection method is of significant importance for enabling early surveillance and precise prevention and control of *S. agalactiae* infection [5,6].

With the advancement of science and technology, particularly in the fields of molecular biology and immunology, the detection methods for *S. agalactiae* have been continuously refined and improved. Currently, various diagnostic techniques, such as quantitative PCR (qPCR) and multiplex PCR, are widely used for the rapid identification and quantitative analysis of *S. agalactiae*. These approaches offer high sensitivity and efficiency, enabling prompt results that assist dairy farmers in implementing timely interventions to prevent the further spread of infection [5,7]. However, these detection methods still encounter certain challenges in clinical applications, including limitations related to sensitivity, specificity, and cost-effectiveness. In some instances, existing diagnostic techniques may fall short of achieving the desired concordance, potentially resulting in missed or incorrect diagnoses [8,9]. In addition, some methods may not be well suited for rapid on-site screening in resource-limited settings. Therefore, developing rapid detection methods for *S. agalactiae* that are highly sensitive, highly specific, and cost-effective is particularly important. This not only helps improve the diagnostic efficiency of mastitis but also provides a reference for the rational use of antibiotics, thereby reducing the emergence of antibiotic-resistant strains [3,8].

In recent years, Recombinase Polymerase Amplification (RPA) has attracted widespread attention due to its rapid reaction time (typically 20–30 min), operation under isothermal conditions (37–42 °C), and low dependence on specialized equipment [10]. Compared with the traditional PCR method, RPA not only reduces the detection time but is also well-suited for application in resource-limited settings [11]. On the other hand, the PfAgo (*Pyrococcus furiosus* Argonaute) protein, derived from thermophilic archaea, possesses programmable single-stranded DNA cleavage activity. The integration of specifically designed guide DNA (gDNA) and molecular beacons enables the precise detection of nucleic acids and targeted cleavage of DNA [12,13]. The nucleic acid cleavage activity of the PfAgo protein offers significant advantages for RPA-based detection methods, particularly in applications requiring high specificity. Studies have demonstrated that the integration of PfAgo and RPA has been effectively utilized for the rapid detection of various pathogens. For instance, an RPA-PfAgo-based assay has been developed for rapid detection of *Salmonella* spp. in food safety applications, supporting the feasibility of combining isothermal amplification with PfAgo-mediated programmable cleavage for pathogen detection [14]. Similarly, the detection of antibiotic resistance genes has also highlighted the robust capabilities of this technology, enabling rapid and accurate on-site analysis [15].

Fluorescence-based readouts provide sensitive and instrument-assisted detection, making them suitable for laboratory-based analysis and result confirmation [16]. In contrast, lateral flow immunochromatographic assays offer a simple visual readout, rapid operation, and minimal equipment requirements, which makes them attractive for field application and use in resource-limited settings [17]. Therefore, integrating these two complementary readout formats into the RPA–PfAgo framework may improve both analytical flexibility and practical applicability for *S. agalactiae* detection. Several genes, including 16S rRNA, *cfb*, *sip*, *scp*B, and *cps*, have been used as molecular targets for *S. agalactiae* detection. The 16S rRNA gene is useful for bacterial identification but may require sequencing confirmation because of conserved regions shared among closely related bacteria. The *sip* and *scp*B genes are also established targets for *S. agalactiae* detection. In the present study, the cfb gene was selected because it has been widely used in PCR- and qPCR-based detection of *S. agalactiae*. In addition, the standardized qPCR assay used for comparison in this study also targeted *cfb*, allowing direct qualitative comparison between the newly developed RPA-PfAgo assays and an established molecular method [18,19,20]. Based on these considerations, the present study selected the *cfb* gene of *S. agalactiae* as the detection target and developed an RPA-PfAgo assay framework for pathogen identification. Specific RPA primers were designed and screened to establish the basic amplification system, followed by the development of two complementary readout formats, namely RPA-PfAgo-RTF and RPA-PfAgo-LFD. The analytical performance of the two methods was evaluated and compared with qPCR, and further validated using 153 field milk samples collected from five regions. Rather than introducing the general concept of combining RPA with PfAgo, this study extends that strategy to the detection of *S. agalactiae* in the context of bovine mastitis and provides a practical basis for rapid pathogen screening in veterinary diagnostics.

## 2. Materials and Methods

### 2.1. Bacterial Strains

*S. agalactiae* (ATCC51487) and *Streptococcus dysgalactiae* (*S. dysgalactiae*, BNCC385341) were purchased from BNCC Bio (Zhengzhou, China). *Staphylococcus aureus* (*S. aureus*, ATCC49525), *Streptococcus uberis* (*S. uberis*, ATCC700407), *Staphylococcus chromogenes* (*S. chromogenes*, ATCC43764), *Salmonella* (CVCC541), *Listeria monocytogenes* (*L. monocytogenes*, isolated strain), *Glaesserella parasuis* (GPS, isolated strain), *Pasteurella multocida* (Pm, C44-1), *Aeromonas hydrophila* (*A. hydrophila*, CVCC4002), and enteropathogenic *Escherichia coli* (*E. coli*, isolated strain) were all preserved by the Department of Preventive Veterinary Medicine at the Henan Institute of Science and Technology.

### 2.2. Reagents

The bacterial genomic DNA kit and PfAgo were purchased from Absin (Shanghai, China) Biotechnology Co., Ltd. The TwistAmp Basic kit was obtained from TwistDx (Cambridge, UK). The PCR Master Mix, plasmid DNA extraction kit, and 4S GelRed nucleic acid staining solution were supplied by Sangon Biotech (Shanghai, China) Co., Ltd. The lateral flow test strips were acquired from Milenia Biotec GmbH (Giessen, Germany), and the gel recovery kit was sourced from Servicebio Biotechnology (Wuhan, China) Co., Ltd. The 20% TBE-PAGE gel preparation solution was purchased from Coolaber Technology (Beijing, China) Co., Ltd. BHI, TSB, and LB media were obtained from Solarbio Technology (Beijing, China) Co., Ltd. TAE (50×) was procured from Beyotime Biomedical Technology (Shanghai, China) Co., Ltd., the 1000 bp DNA marker was acquired from BaKaRa Medical Biology Technology (Beijing, China) Co., Ltd. LAR AGAROSE/agarose was obtained from baygene Biotechnology (Shanghai, China) Co., Ltd.

### 2.3. Preparation of Bacterial DNA

The frozen bacterial samples were thawed and subsequently streaked onto solid growth medium for culturing. Individual colonies were then selected and further cultured in liquid medium. Specifically, BHI (Brain Heart Infusion) medium was used for the cultivation of *Listeria*. TSB (Tryptic Soy Broth) medium was employed for the culture of *S. agalactiae*, Pm, and GPS. LB (Luria–Bertani) medium was used for the growth of *A. hydrophila*, *E. coli*, *S. aureus*, and *Salmonella*. Bacterial genomic DNA was extracted from cultures in the logarithmic growth phase using a commercial bacterial genomic DNA extraction kit.

### 2.4. RPA Primers Design

The conserved gene *cfb* of *S. agalactiae* (Accession Number: JQ289582.1) was selected as the target. Primer design was conducted using Primer 5.0 software, following the guidelines provided by the TwistAmp DNA Amplification Kit (Cambridge, UK). A total of seven primer pairs were designed (Table 1) and subsequently synthesized by Sangon Biotech (Shanghai) Co., Ltd.

According to the instructions provided in the TwistAmp^®^ RPA Kit (TwistDx, Cambridge, UK), a 50 μL reaction system was prepared for the RPA reaction. This system included 29.5 μL of reaction buffer, 2.5 μL of 280 mM magnesium acetate, 2.4 μL of 10 μM forward and reverse primers, 2 μL of DNA template, and 11.2 μL of deionized water. The components were gently mixed and briefly centrifuged to ensure homogeneity. The reaction mixture was then incubated at 39 °C for 30 min. The resulting amplification products were analyzed using a 2% agarose gel, and the most effective primer pair was selected based on the electrophoresis results.

### 2.5. Basic-RPA Assay Setup and Optimization Procedures

To improve the amplification efficiency, the reaction temperatures (25 °C, 30 °C, 35 °C, 37 °C, 39 °C, and 45 °C) and reaction times (10 min, 20 min, 25 min, 30 min, and 35 min) were systematically optimized. The genomic DNA of GBS was used as the positive control, while ten non-target bacterial species, including *L*. *monocytogenes*, GPS, Pm, *Salmonella*, *A*. *hydrophila*, *E*. *coli*, *S*. *aureus*, *S*. *chromogenes*, *S*. *uberis* and *S. dysgalactiae* were used to evaluate analytical specificity. Subsequently, amplification reactions were conducted using the optimal primer pair identified in the preliminary screening, along with the optimized time and temperature parameters of the Basic-RPA reaction system. The reaction products were analyzed via agarose gel electrophoresis to assess potential cross-reactivity with the ten non-target bacterial species, thereby validating the specificity of the detection method. Additionally, the *S. agalactiae* DNA template was serially diluted to eight concentrations ranging from 10^0^ to 10^−5^ ng/µL. The detection limit of the system was determined by evaluating the presence or absence of amplification bands at each dilution level.

### 2.6. Verification of PfAgo Cleavage Activity and Screening of Candidate gDNAs

To verify whether the PfAgo protein can efficiently cleave ssDNA, six gDNA sequences, each 16 nt in length, were designed based on the conserved *cfb* gene sequence of *S. agalactiae*. These gDNAs were used to mediate the cleavage of ssDNA by PfAgo, and the resulting products were analyzed using 20% TBE-PAGE electrophoresis. The most effective gDNA was selected through this screening process. Based on the selected gDNA sequences, two additional gDNA primers targeting the *cfb* gene of *S. agalactiae* were designed, and corresponding molecular beacons were also developed. All gDNA and molecular beacon sequences were synthesized for subsequent detection of the *cfb* gene. The molecular beacon used for fluorescence detection was modified with FAM at the 5′ end and BHQ1 at the 3′ end. For the lateral flow strip detection method, the molecular beacon was similarly labeled with FAM at the 5′ end and biotin at the 3′ end (Table 2).

### 2.7. RPA-PfAgo-RTF Assay Setup and Optimization Procedures

The conserved gene *cfb* was cloned into the recombinant plasmid pGEM-T Easy (Promega, Beijing), and the resulting positive plasmid was designated as pGEM-T-cfb and used as a DNA template standard. Following determination of the plasmid concentration, the copy number was calculated using the following formula: Copy number (copies/μL) = [6.02 × 10^23^ × plasmid concentration (ng/μL)]/[DNA length (bp) × 660 × 10^9^]. Basic-RPA reactions were performed under optimized conditions at a 1.5-fold scale. Subsequently, 2 μL of the RPA product was added to the PfAgo reaction mixture. The reaction mixture contained 2 μL of Zg5 (10 μM), 2 μL of Zg5-2 (10 μM), 2 μL of Zg5-3 (10 μM), 1 μL of molecular beacon (MB) (10 μM), 1 μL of PfAgo endonuclease, 1 μL of MnCl_2_ (40 mM), and 2.5 μL of 10× reaction buffer. The final volume was adjusted to 25 μL using enzyme-free water to construct the RPA-PfAgo-RTF reaction system. The reaction was carried out in a fluorescence quantitative PCR instrument at a constant temperature of 95 °C for a duration of 50 min. To optimize the reaction conditions, various concentrations of MnCl_2_ (0.5, 1, 1.6, 2, 2.4, and 3.2 mM), probe (0 μM, 6 μM, 7 μM, 8 μM, 9 μM, and 10 μM), and gDNA (0.2 μM, 0.5 μM, 0.8 μM, 1.2 μM, and 1.5 μM) were tested, along with different reaction temperatures (89 °C, 92 °C, 95 °C, and 98 °C) and reaction times (10 min, 20 min, 30 min, 40 min, 50 min, and 60 min). Based on fluorescence signal intensity and reaction stability, 50 min was selected as the final optimized PfAgo reaction time for subsequent RPA-PfAgo-RTF assays. To establish a reliable standard system and accurately distinguish positive and negative samples based on the fluorescence detection method mediated by PfAgo protein, this study employed a fluorescence quantitative PCR instrument to monitor the fluorescence signals of the amplification products. Multiple replicate experiments were conducted using low-copy-number plasmids to determine the criteria for identifying positive samples using this fluorescence detection method.

### 2.8. Establishment of the RPA-PfAgo-LFD Assay

The RPA-PfAgo detection system was integrated with lateral flow dipstick (LFD) technology to establish a visual detection assay, termed RPA-PfAgo-LFD. In this system, the original RTF-MB probe from the RPA-PfAgo-RTF assay (Section 2.7) was replaced with an LFD-MB probe. Interpretation of results followed these criteria: cleavage of the probe in positive samples generated visible bands at both the test (T) and control (C) lines, whereas negative samples exhibited a band only at the control line due to uncleaved probes. Absence of bands on both lines indicated an invalid test.

### 2.9. Evaluation of Specificity and Sensitivity for the RPA-PfAgo-RTF and RPA-PfAgo-LFD

The pGEM-T-cfb plasmid was serially diluted tenfold from 1 × 10^6^ to 1 × 10^0^ copies/µL and subjected to isothermal amplification via the optimized RPA protocol. Amplification products were evaluated using the optimized RPA-PfAgo-RTF and RPA-PfAgo-LFD assays to determine their detection sensitivities. Specificity was assessed using genomic DNA from GBS, *L. monocytogenes*, GPS, Pm, *Salmonella*, *A. hydrophila*, *E. coli*, *S. aureus*, *S. chromogenes*, *S. uberis* and *S. dysgalactiae* as templates.

### 2.10. Bacteriological Culture and qPCR Assay

Bacteriological culture was used as the conventional reference comparator for comparison of field milk sample results. Culture-based isolation and identification of *S. agalactiae* in bovine mastitis milk samples were performed according to the agricultural industry standard NY/T 2962—2016 [21]. Positive and negative culture results were used for calculating agreement indices between culture and molecular assays.

In parallel, a qPCR assay was used as the reference molecular method for comparison with the two RPA-PfAgo assays. The qPCR assay targeted the *cfb* gene of *S. agalactiae* and was performed according to SN/T 5887—2025 [22]. In this standardized method, *cfb* is used as the target gene for *S. agalactiae* detection. Interpretation of qPCR results followed the criteria of the standard: Ct values ≤ 35.0 were considered positive, Ct values ≥ 40.0 were considered negative, and samples with Ct values > 35.0 but <40.0 were retested; if the repeat result remained <40.0, the sample was considered positive; otherwise, it was considered negative.

Thus, in this study, bacteriological culture performed according to NY/T 2962—2016 served as the conventional comparator for agreement analysis, whereas qPCR performed according to SN/T 5887—2025 served as the reference molecular method for concordance analysis with RPA-PfAgo-RTF and RPA-PfAgo-LFD [21,22].

### 2.11. Field Milk Samples and Comparative Evaluation

A total of 153 bovine milk samples were collected in 2024 from dairy farms located in five regions of China, including Henan, Liaoning, Heilongjiang, Shandong, and Hebei. These samples were submitted by farm owners for laboratory testing. Samples were included when they had clear sample identification, sufficient volume for bacteriological culture and molecular assays, and available clinical or California Mastitis Test (CMT)-based classification information. Samples with insufficient volume, visible contamination, leakage during transport, unclear labeling, incomplete essential information, or duplicate submissions from the same sampling event were excluded.

Clinical mastitis samples were defined as milk samples from cows showing visible milk abnormalities and/or udder clinical signs. Subclinical mastitis samples were identified based on positive CMT results in the absence of obvious clinical signs. Healthy milk samples were obtained from cows without clinical signs and with negative CMT results. According to these criteria, the samples were classified into 67 clinical mastitis samples, 61 subclinical mastitis samples, and 25 healthy milk samples. Somatic cell count data were not available for all submitted samples; therefore, CMT results together with clinical records were used for sample classification in this study. When available, quarter-level sampling information and recent antimicrobial treatment history were recorded; however, complete quarter-level and antimicrobial treatment records were not available for all submitted samples.

The regional distribution was as follows: Henan, 19 samples (12 clinical mastitis and 7 subclinical mastitis); Liaoning, 31 samples (9 clinical mastitis, 12 subclinical mastitis, and 10 healthy); Heilongjiang, 45 samples (21 clinical mastitis, 16 subclinical mastitis, and 8 healthy); Shandong, 33 samples (15 clinical mastitis, 11 subclinical mastitis, and 7 healthy); and Hebei, 25 samples (10 clinical mastitis and 15 subclinical mastitis).

For molecular detection, casein and fat were removed from milk samples using the citric acid method before DNA extraction. DNA was then extracted using a commercial DNA extraction kit. DNA concentration and purity were measured using an ultra-micro nucleic acid and protein analyzer, and DNA samples were stored at −80 °C until use. The extracted DNA was used for qPCR, RPA-PfAgo-RTF, and RPA-PfAgo-LFD. Comparative evaluation of the four methods, including bacteriological culture, qPCR, RPA-PfAgo-RTF, and RPA-PfAgo-LFD, was performed using the same field milk sample set.

### 2.12. Statistical Analysis

Agreement analysis was performed on the basis of paired sample-by-sample results for all 153 milk samples. Using bacteriological culture as a conventional comparator, positive agreement, negative agreement, positive predictive agreement (PPA), negative predictive agreement (NPA), and Kappa values were calculated for qPCR, RPA-PfAgo-RTF, and RPA-PfAgo-LFD. Using the standardized real-time PCR assay as the reference molecular method, overall concordance between qPCR and each RPA-PfAgo assay was evaluated on a sample-by-sample basis. Positive and negative counts for each method were summarized for the field validation dataset.

## 3. Results

### 3.1. Design and Screening of RPA Primers

**Figure 1:**
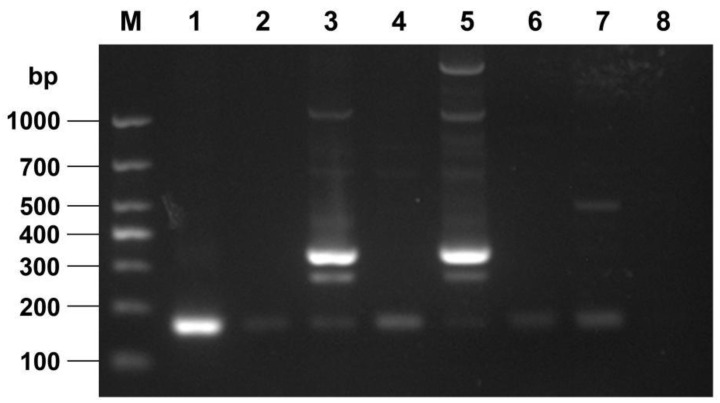
Design and screening of RPA primers targeting the *cfb* gene of *Streptococcus agalactiae*. Amplification products generated by seven candidate RPA primer pairs were analyzed by agarose gel electrophoresis. M, 1000 bp DNA ladder. Lanes 1–8 correspond to primer pairs Cfb166, Cfb275, Cfb164, Cfb219, Cfb165, Cfb218, Cfb288, and the no-template control (NTC), respectively. The primer pair Cfb166 produced a clear target band and was selected for subsequent experiments.

According to the RPA primer design principles, seven pairs of *S. agalactiae*-specific primers were designed targeting the conserved *cfb* gene of the organism. Amplification was performed using the constructed pGEM-T-cfb plasmid as a template, followed by agarose gel electrophoresis for product evaluation. Agarose gel electrophoresis revealed that the primer pair Cfb166F/Cfb166R generated a distinct target amplicon, whereas no amplification was observed in the negative control (Figure 1). Consequently, Cfb166F/Cfb166R was selected for subsequent assays.

### 3.2. Establishment and Optimization of the Basic-RPA Assay

**Figure 2:**
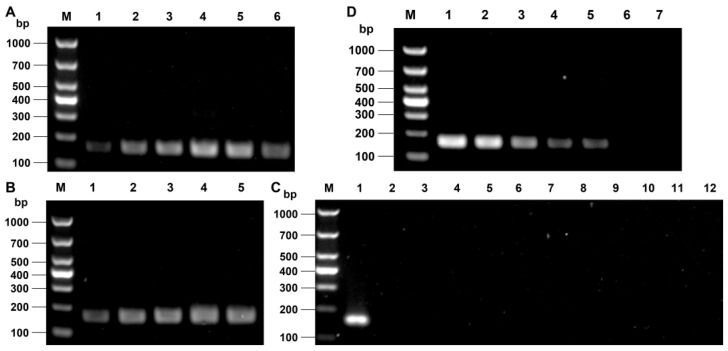
Establishment and optimization of Basic-RPA assay. (**A**) Optimization of reaction temperature for the Basic-RPA assay. M, DL1000 DNA marker. Lanes 1–6 represent amplification products obtained at 25, 30, 35, 37, 39, and 45 °C, respectively. (**B**) Optimization of reaction time for the Basic-RPA assay. M, DL1000 DNA marker. Lanes 1–5 represent amplification products obtained after incubation for 10, 20, 25, 30, and 35 min, respectively. (**C**) Analytical specificity of the Basic-RPA assay. M, DL1000 DNA marker. Lanes 1–12 represent GBS, *L. monocytogenes*, GPS, Pm, *Salmonella*, *A. hydrophila*, *E. coli*, *S. aureus*, *S. chromogenes*, *S. uberis*, *S. dysgalactiae*, and the no-template control (NTC), respectively. (**D**) Amplification performance of the Basic-RPA assay. M, DL1000 DNA marker. Lanes 1–7 represent serial ten-fold dilutions of the pGEM-T-cfb plasmid template at 1 × 10^0^, 1 × 10^−1^, 1 × 10^−2^, 1 × 10^−3^, 1 × 10^−4^, and 1 × 10^−5^ ng/µL, and the NTC, respectively.

To achieve the optimal Basic-RPA efficiency, both reaction time and temperature were systematically optimized. With a reaction time of 30 min and a primer concentration of 10 μmol/L, amplification was evaluated across a temperature range of 30~45 °C. Target amplicons were detected at all tested temperatures, and clear amplification bands were observed at 37 °C (Figure 2A and Supplementary Figure S1A), which was therefore selected as the optimal reaction temperature. Subsequently, with temperature fixed at 37 °C and primer concentration at 10 μmol/L, amplification was assessed at various reaction times. Amplicons were detected from 10 to 35 min, and a clear amplification band was observed at 30 min (Figure 2B and Supplementary Figure S1B). Therefore, 30 min was selected as the reaction time for subsequent experiments.

The specificity of the Cfb166F/Cfb166R primers was assessed using the optimized Basic-RPA assay against ten non-target bacterial pathogens. As shown in Figure 2C, amplification yielded a single, distinct band exclusively in the positive control, with no amplification products detected for the non-target species or the no-template control, confirming the absence of detectable cross-reactivity under the tested conditions. To assess the amplification performance of the Basic-RPA step, serially diluted *S. agalactiae* DNA was used as the template. As shown in Figure 2D, a visible amplification band was detected down to 1 × 10^−3^ ng/µL under the tested gel-based conditions.

### 3.3. Verification of PfAgo Cleavage Activity and Screening of Candidate gDNA

**Figure 3:**
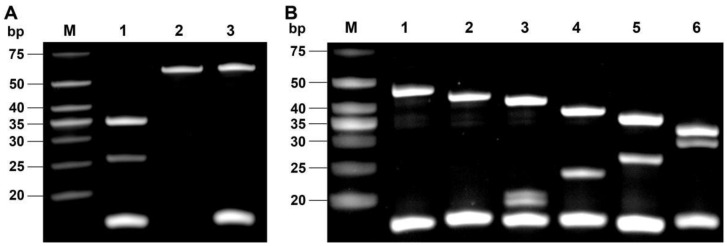
Verification of PfAgo cleavage activity and screening of candidate gDNAs. (**A**) Verification of the cleavage activity of PfAgo using the target ssDNA substrate in the presence or absence of guide DNA. (**B**) Screening of six candidate gDNAs for PfAgo-mediated cleavage of the target ssDNA. Cleavage products were analyzed by 20% TBE-PAGE. M, DNA marker. Lanes 1–6 in panel B represent the cleavage products generated using the six candidate gDNAs, respectively.

As shown in Figure 3A, PfAgo cleaved the target DNA into two fragments of distinct sizes, confirming its single-stranded DNA (ssDNA) cleavage activity. In the experimental setup, six designed guide DNA (gDNA) sequences were individually introduced into the PfAgo reaction system. Following the reaction, 20% TBE-PAGE gel electrophoresis was conducted to assess cleavage efficiency. The results revealed notable variation in the efficacy of different gDNA sequences in mediating ssDNA cleavage by PfAgo. Among the six gDNA primers tested, Zg5 exhibited the highest cleavage efficiency (Figure 3B). Based on Zg5, two additional gDNA primers were designed, and corresponding molecular beacons were developed (Table 2) to support further in-depth investigations in subsequent experiments.

### 3.4. Establishment and Optimization of the RPA-PfAgo-RTF Assay

The RPA-PfAgo-fluorescence reaction system consisted of 2 µL of gDNA (1.5 µM), 1–2 units of PfAgo protein, 2.5 µL of reaction buffer, 1 µL of Mn^2+^ (2.4 mM), 1.5 µL of molecular beacon (9 µM), and 2 µL of DNA template, with ddH_2_O added to a final volume of 25 µL.

**Figure 4:**
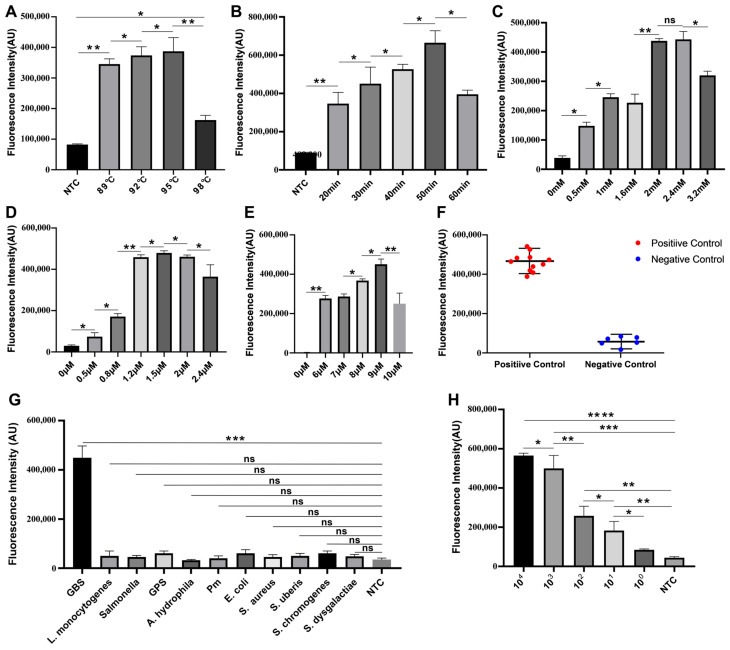
Establishment and optimization of RPA-PfAgo-RTF assay. (**A**) Optimization of reaction temperature for the RPA-PfAgo-RTF assay. Fluorescence intensity was measured at 89, 92, 95, and 98 °C, with the NTC included as the negative control. (**B**) Optimization of reaction time for the RPA-PfAgo-RTF assay. Fluorescence signals were measured after 20, 30, 40, 50, and 60 min of incubation. (**C**) Optimization of Mn^2+^ concentration in the RPA-PfAgo-RTF reaction system. (**D**) Optimization of gDNA concentration in the RPA-PfAgo-RTF reaction system. (**E**) Optimization of molecular beacon (MB) concentration in the RPA-PfAgo-RTF reaction system. (**F**) Determination of the fluorescence threshold for positivity judgment. Red dots represent fluorescence values obtained from positive controls containing the target *cfb* template, and blue dots represent fluorescence values obtained from negative controls without template (NTC). Horizontal lines indicate the distribution range of the fluorescence signals in each group. Based on repeated measurements of positive and negative controls under optimized conditions, a fluorescence intensity threshold of 100,000 AU was adopted in this study for distinguishing positive and negative reactions. (**G**) Analytical specificity of the RPA-PfAgo-RTF assay. Genomic DNA from *S. agalactiae* was used as the positive control, while genomic DNA from non-target bacterial species and the NTC were used as negative controls. (**H**) Analytical sensitivity of the RPA-PfAgo-RTF assay using serial dilutions of the pGEM-T-cfb plasmid template. NTC served as the negative control. Data in panels (**A**–**E**,**G**,**H**) are presented as mean ± SEM from three independent experiments. AU, arbitrary fluorescence units. Statistical significance was defined as follows: ns, not significant; *, *p* < 0.05; **, *p* < 0.01; ***, *p* < 0.001; ****, *p* < 0.0001.

To establish suitable reaction conditions for the RPA-PfAgo-RTF assay, reaction temperature, reaction time and key reaction components, including MnCl_2_, gDNA, and MB probe concentrations, were evaluated systematically. Under initial conditions (40 min, 2 mM MnCl_2_, 1.2 μM gDNA, 8 μM MB), amplification was tested across temperatures ranging from 89 °C to 98 °C. Detectable fluorescence signals were observed over this temperature range, and the signal at 95 °C was clearly distinguishable under the tested conditions (Figure 4A). Therefore, 95 °C was selected for subsequent experiments. Using 95 °C as the reaction temperature, different reaction times were further evaluated. Detectable fluorescence signals were observed after approximately 20 min of reaction, and a clear signal was obtained at 50 min (Figure 4B). Therefore, 50 min was selected as the final PfAgo reaction time for subsequent experiments. The Mn^2+^ concentration, gDNA concentration, and MB probe concentration were subsequently optimized under the selected temperature and time conditions. Based on the observed fluorescence results under the tested conditions, 2.4 mM Mn^2+^, 1.5 μM gDNA, and 9 μM MB probe were selected for the final RPA-PfAgo-RTF reaction system (Figure 4C–E). Based on repeated measurements of positive and negative controls under the optimized conditions, a fluorescence threshold of 100,000 AU was empirically set for this study [23]. Values below this threshold were considered negative, while values above were considered positive (Figure 4F).

Analytical specificity was further evaluated using the same panel of ten non-target bacterial species. As shown in Figure 4G, the *S. agalactiae* group produced a fluorescence signal exceeding 400,000 AU, whereas all non-target bacteria generated signals comparable to the negative control and well below the cutoff threshold. These results demonstrate that the RPA-PfAgo-RTF assay specifically and accurately detects the target nucleic acid with high specificity. Sensitivity evaluation using ten-fold serial dilutions of the pGEM-T-cfb plasmid (10^6^ to 10^0^ copies/μL) under optimized conditions demonstrated a detection limit of 10 copies/μL (Figure 4H).

### 3.5. Analytical Specificity and Analytical Sensitivity of the RPA-PfAgo-LFD Assay

**Figure 5:**
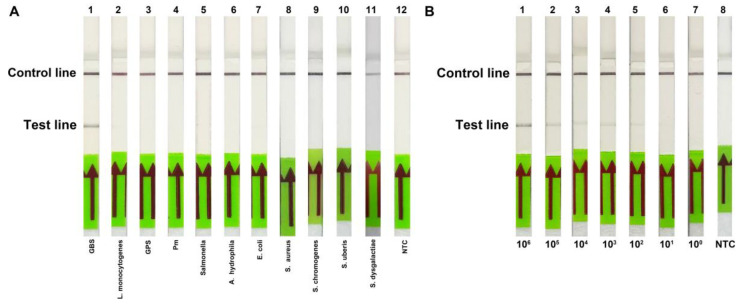
Analytical specificity and analytical sensitivity of the RPA-PfAgo-LFD assay. (**A**) Analytical specificity of the RPA-PfAgo-LFD assay. Strips 1–12 represent GBS, *L. monocytogenes*, GPS, Pm, *Salmonella*, *A. hydrophila*, *E. coli*, *S. aureus*, *S. chromogenes*, *S. uberis*, *S. dysgalactiae*, and the NTC, respectively. (**B**) Analytical sensitivity of the RPA-PfAgo-LFD assay. Strips 1–8 represent seven serially diluted pGEM-T-cfb plasmid DNA concentrations ranging from 1 × 10^6^ to 1 × 10^0^ copies/μL and the NTC, respectively. For the lateral flow dipstick readout, the upper band indicates the control line (C line) and the lower band indicates the test line (T line). The presence of both the C line and T line was interpreted as a positive result, whereas the presence of only the C line was interpreted as a negative result. Absence of the C line was considered an invalid test.

Specificity testing with genomic DNA from GBS, *L. monocytogenes*, GPS, Pm, *Salmonella*, *A. hydrophila*, *E. coli*, *S. aureus*, *S. uberis*, *S. chromogenes* and *S. dysgalactiae* demonstrated positive detection exclusively for *S. agalactiae*, with no cross-reactivity observed (Figure 5A), confirming high assay specificity. The sensitivity of the RPA-PfAgo-LFD assay was evaluated using ten-fold serial dilutions of the pGEM-T-cfb plasmid (1 × 10^6^ to 1 × 10^0^ copies/μL) under optimized conditions. Test line intensity on lateral flow strips decreased with target concentration, with a detection limit of 100 copies/μL, slightly lower than that of the fluorescence assay (Figure 5B).

### 3.6. Multiregional Field Validation of qPCR, RPA-PfAgo-RTF, and RPA-PfAgo-LFD Using Bovine Milk Samples

**Figure 6:**
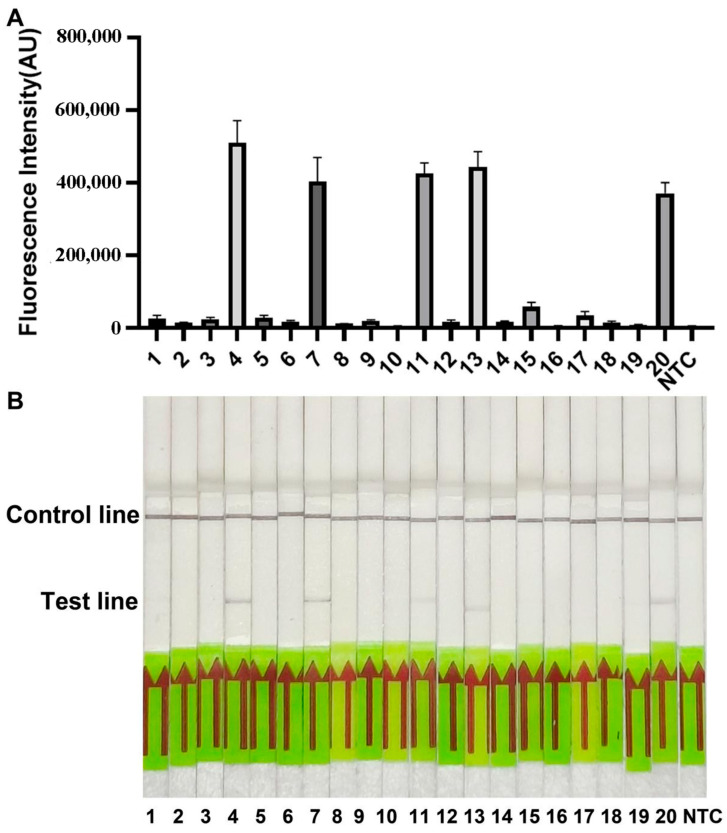
Representative detection results of *Streptococcus agalactiae* in field milk samples using RPA-PfAgo-RTF and RPA-PfAgo-LFD. (**A**) Fluorescence signal intensities of 20 representative milk samples detected by RPA-PfAgo-RTF. Samples 4, 7, 11, 13, and 20 were positive. A fluorescence intensity above 100,000 AU was considered positive. (**B**) Lateral flow dipstick results for the same 20 representative milk samples detected by RPA-PfAgo-LFD. Samples 4, 7, 11, 13, and 20 were positive. The presence of both C and T lines indicates a positive result, whereas the presence of only the C line indicates a negative result.

Field validation included 153 bovine milk samples collected in 2024 from five dairy-farming regions in China, including 67 clinical mastitis samples, 61 subclinical mastitis samples, and 25 healthy milk samples (Supplementary Table S1), thereby providing broad geographic and clinical coverage for assay evaluation. Across all 153 samples, RPA-PfAgo-RTF and RPA-PfAgo-LFD were completely concordant with the qPCR assay, indicating that the newly developed assays achieved the same qualitative detection outcome as the reference molecular method in practical milk-sample testing. When bacteriological culture was used as a conventional comparator, qPCR, RPA-PfAgo-RTF, and RPA-PfAgo-LFD all showed 100.00% positive agreement, 90.91% negative agreement, and a Kappa value of 0.733 (Table 3); representative detection results are shown in Figure 6. Notably, the molecular assays identified 33 positive samples, whereas culture identified 21, including 15 versus 10 positives in clinical mastitis samples and 18 versus 11 positives in subclinical mastitis samples. In addition, all 25 healthy milk samples were negative by all four methods, and the uniformly negative results observed in the healthy group further support the specificity of the molecular assays in field samples.

## 4. Discussion

*S*. *agalactiae* is one of the primary pathogens responsible for mastitis in dairy cattle, resulting in substantial economic losses to the dairy industry and negatively affecting both milk yield and dairy product quality. Research indicates that mastitis caused by *S. agalactiae* accounts for 30% to 70% of all mastitis cases in dairy cows [24]. Beyond its impact on animal health, *S. agalactiae* poses a public health risk due to potential transmission through dairy products. The recent expansion and intensification of dairy farming have altered the epidemiology of *S. agalactiae* infections, posing new challenges for effective control [25,26]. Conventional diagnostic methods are labor-intensive, time-consuming, and reliant on specialized equipment, hindering rapid detection. Thus, developing a highly sensitive, specific, and instrument-independent assay for early detection and control of *S. agalactiae* is imperative. Such advancements are vital for improving bovine health, milk production, and the sustainability of the dairy industry.

Several target genes, including 16S rRNA, *cfb*, *scp*B, *cps*, and *sip*, have been used for molecular detection of *S. agalactiae* [27,28,29]. Although 16S rRNA is widely used for bacterial classification, its conserved regions among closely related streptococci may require sequencing confirmation and make primer design more challenging. Species-associated targets, such as *cfb*, *sip*, and *scp*B, are therefore more appropriate for targeted *S. agalactiae* detection. Among them, *cfb* is conserved and specific across *S. agalactiae* strains from different serotypes and host origins, and cfb-based PCR or qPCR assays have been successfully applied to bovine mastitis-associated isolates and milk samples [29,30]. Accordingly, *cfb* was selected in this study because it combines diagnostic specificity, relevance to bovine milk testing, and consistency with the standardized qPCR assay used as the reference molecular comparator. In this study, the conserved *cfb* gene of *S. agalactiae* was selected as the target, and the primer pair Cfb166F/Cfb166R was identified as the optimal RPA primer set through design and screening. The development of RPA primers must consider both the conservation of the target region and the efficiency of amplification, as the success of this step directly influences the sensitivity and specificity of the subsequent detection system [11]. Experimental results showed that this primer set yielded no amplification in negative controls and exhibited the best performance among seven candidate pairs (Figure 1), with no nonspecific bands detected in assays against various non-target bacteria (Figure 2C). These findings align with previous reports emphasizing that highly specific primers are critical for reliable RPA assays, thereby laying a solid foundation for the stability of subsequent PfAgo-based detection methods [31].

PfAgo, a thermophilic archaeal protein, exhibits temperature-dependent ssDNA cleavage [32]. This study demonstrated efficient cleavage by PfAgo, with Zg5 identified as the optimal gDNA guide (Figure 3B), supporting subsequent fluorescence and lateral flow assays. Unlike Cas nucleases, PfAgo requires no PAM sequence and allows programmable cleavage via gDNA design, enhancing target flexibility in molecular diagnostics [33]. Specificity assays showed strong fluorescence in positive controls and negligible signals in non-target pathogens, confirming precise target recognition and minimizing nonspecific binding. This specificity ensures assay reliability and reduces false positives, critical for clinical diagnostics and pathogen screening. By combining PfAgo with RPA, this study integrated efficient cleavage with isothermal amplification, enhancing both specificity and sensitivity. Targeting the *S. agalactiae cfb* gene, six gDNAs were designed and screened, and reaction conditions—including temperature, time, and Mn^2+^, gDNA, and molecular beacon concentrations—were optimized. Optimal conditions were determined as 37 °C and 30 min for Basic-RPA, achieving a detection limit of 10^−3^ ng/μL (Figure 4H). This sensitivity surpasses that reported in some RPA-based pathogen detection studies [16], indicating that the method is well-suited for rapid early clinical detection.

The isothermal RPA-PfAgo system eliminates thermal cycling, enabling use with simplified, portable devices, which is advantageous for point-of-care testing in remote areas. The developed RPA-PfAgo fluorescence assay achieved a detection limit of 10 copies/μL at 95 °C within 50 min with high specificity (Figure 4G,H) and superior sensitivity compared to the LFD assay (100 copies/μL) (Figure 5B). While fluorescence detection offers objective signal acquisition and higher analytical sensitivity, it requires a fluorescence detection instrument. In contrast, the LFD format enables visual interpretation and therefore simplifies result readout [34]. Although the RPA can be completed at low temperatures, the PfAgo-mediated detection requires 95 °C, resulting in an assay workflow that is not entirely equipment-free. Both methods specifically distinguished *S. agalactiae* from non-target bacteria, indicating that PfAgo-mediated secondary recognition effectively reduces nonspecific amplification [35]. Field validation showed complete agreement between qPCR, RPA-PfAgo-RTF, and RPA-PfAgo-LFD across all 153 bovine milk samples, and representative detection results are shown in Figure 6. These findings support the qualitative concordance of the RPA-PfAgo assays with qPCR in the tested field sample set and suggest their potential value for molecular detection of *S. agalactiae* in bovine milk.

From an application perspective, the dual-readout design provides complementary options for different testing scenarios. The RPA-PfAgo-RTF assay is more suitable for laboratory-based testing or confirmatory analysis because it provides higher analytical sensitivity and objective fluorescence signals, but it requires a fluorescence detection instrument. In contrast, the RPA-PfAgo-LFD assay enables visual result interpretation and may be more convenient for field screening when DNA extraction and a compact heating device are available. The current workflow requires approximately 30 min for RPA followed by 50 min for PfAgo-mediated detection, excluding DNA extraction. Therefore, although the LFD format reduces dependence on fluorescence detection equipment, the assay should be considered a simplified visual molecular detection format rather than a fully equipment-free point-of-care test. The per-test cost was not formally calculated in this study and may vary depending on reagent source and testing scale. A comparison of the present RPA-PfAgo platform with LAMP, conventional RPA-LFD, and CRISPR-Cas-based assays is summarized in Supplementary Table S2.

Field validation further supported the practical performance of the developed platform. Both RPA-PfAgo-RTF and RPA-PfAgo-LFD were completely concordant with the standardized qPCR assay across all 153 bovine milk samples, indicating equivalent qualitative detection outcomes relative to the reference molecular method used in this study. When bacteriological culture was used as a conventional comparator, all three molecular assays showed 100.00% positive agreement, 90.91% negative agreement, and a Kappa value of 0.733. These findings highlight the complementary roles of molecular assays and bacteriological culture. The RPA-PfAgo assays provide rapid and sensitive detection of *S. agalactiae* DNA and are useful for screening and surveillance, particularly when rapid decision-making is required. In contrast, bacteriological culture remains important for confirming viable bacteria and obtaining isolates for further characterization, such as antimicrobial susceptibility testing. Therefore, the additional molecular-positive/culture-negative samples should not be interpreted simply as false positives or as definitive viable infections, but rather as molecular evidence that should be considered together with clinical signs, herd history, and culture results. In addition, all 25 healthy milk samples were negative by all four methods, which further supports the practical specificity of the molecular assays in this sample set.

Although the present findings are encouraging, several limitations should be acknowledged. First, although samples were collected from five dairy-farming regions, the field validation cohort remained moderate in size, with only 33 qPCR-positive samples among the 153 tested samples; therefore, larger independent cohorts with more positive samples are still needed to further evaluate assay robustness across different herds, bacterial loads, and management conditions. Second, the assay was developed using a single target gene, and additional evaluation across a broader range of strains and field conditions would further strengthen its generalizability. Third, discrepant samples between culture and molecular assays were not further resolved by sequencing or by applying a composite reference framework. In addition, gDNA screening was initially performed using synthetic ssDNA substrates; although the selected gDNA was subsequently evaluated in the complete RPA-PfAgo workflow, direct comparison of PfAgo cleavage efficiency on synthetic ssDNA and RPA amplicons was not performed. Moreover, reaction optimization was conducted using a stepwise strategy rather than factorial or response-surface design; therefore, possible interaction effects among temperature, reaction time, MnCl_2_, gDNA, and molecular beacon concentrations were not fully assessed.

## 5. Conclusions

In summary, the dual-readout RPA-PfAgo platform demonstrated high analytical specificity and sensitivity, complete concordance with standardized qPCR, and a higher positive detection rate than bacteriological culture in field milk samples. The fluorescence format is more suitable for laboratory-based testing or confirmatory analysis, whereas the LFD format offers simplified visual interpretation and may support field screening when DNA extraction and compact heating equipment are available. Together, these findings support the potential of RPA-PfAgo-RTF and RPA-PfAgo-LFD as efficient molecular tools for detecting *S. agalactiae* in bovine milk samples.

## Figures and Tables

**Table 1:** Primers used in the experiments.

Primers	Sequence (5′ → 3′)	Product/bp
Cfb166	F: AAGCTAGTTATTCGCATTTTAGATCCATTT	166
	R: CCAGATTTCCTTATCAAGTTTTGATTTTGT	
Cfb275	F: TGAGAAATATCAAAGATAATGTTCAGGGAA	275
	R: AGCAAATGGATCTAAAATGCGAATAACTAG	
Cfb164	F: TGAGAAATATCAAAGATAATGTTCAGGGAA	164
	R: AATCACATCTGTTAAGGCTTCTACACGACT	
Cfb219	F: ATGTAAATAGTAATAATCAAGCCCAGCAAA	219
	R: CTGTTAAGGCTTCTACACGACTACCAATAG	
Cfb165	F: TATTCGCATTTTAGATCCATTTGCTTCAGT	165
	R: CGTGTATTCCAGATTTCCTTATCAAGTTTT	
Cfb218	F: GTAAATAGTAATAATCAAGCCCAGCAAATG	218
	R: TCTGTTAAGGCTTCTACACGACTACCAATA	
Cfb288	F: TTGGTAGTCGTGTAGAAGCCTTAACAGATG	288
	R: AGCGTGTATTCCAGATTTCCTTATCAAGTT	

**Table 2:** gDNAs and MBs for detecting *Streptococcus agalactiae*.

gDNA/MB/ssDNA	Sequence (5′ → 3′)
Zg1	TAA CTT GAG CTT TAA T
Zg2	CGT TAA CTT GAG CTT T
Zg3	CAT CGT TAA CTT GAG C
Zg4	TTA CAT CGT TAA CTT G
Zg5	CCT TTA CAT CGT TAA C
Zg6	ATG CCT TTA CAT CGT T
Zg5-2	ACC TTT TGT TCT AAT G
Zg5-3	TCA AGT TAA CGA TGT A
bMB	FAM-TTTGTTCTAATGCCTTTACATCGTTAAC-BHQ1
LFD-MB	FAM-CTAATGCCTTTACATC-Biotin
ssDNA	TGATTCAATTAAAGCTCAAGTTAACGATGTAAAGGCATTAGAACAAAAGGTTTTAACTT

**Table 3:** Comparative performance of bacteriological culture, standardized qPCR, RPA-PfAgo-RTF, and RPA-PfAgo-LFD in 153 field milk samples.

Detection Methods	Number of Positive	Number of Negative	Positive agreement (%)	Negative agreement (%)	PPA (%)	NPA (%)	Kappa
Culture	21	132	-	-	-	-	-
qPCR	33	120	100.00	90.91	63.64	100.00	0.733
RPA-PfAgo-RTF	33	120	100.00	90.91	63.64	100.00	0.733
RPA-PfAgo-LFD	33	120	100.00	90.91	63.64	100.00	0.733

## Data Availability

The data presented in this study are available in the article and Supplementary Materials. Further inquiries can be directed to the corresponding authors.

## Supplementary Materials

The following supporting information can be downloaded at: https://www.mdpi.com/article/10.3390/vetsci13060561/s1, Figure S1: Quantitative analysis of Basic-RPA amplification under different temperature and time conditions; Table S1: Regional distribution and clinical classification of the 153 bovine milk samples included in the field validation study; Table S2: Comparison of RPA-PfAgo with other rapid molecular detection methods.

## Abbreviations

The following abbreviations are used in this manuscript:

RPA	recombinase polymerase amplification
gDNAs	guide DNAs
RTF	real-time fluorescence
LFD	lateral flow dipstick
GBS	Group B Streptococcus
qPCR	quantitative PCR
ssDNA	single-stranded DNA
