# Supplementary material for: Development and Application of a Dual-Readout RPA-PfAgo System for Rapid Detection of Streptococcus agalactiae in Bovine Milk
Source: Vet Sci. 2026 Jun 6;13(6):561. doi: 10.3390/vetsci13060561 (PMC13307622; doi:10.3390/vetsci13060561)
Supplement: Supplementary file 1 [file vetsci-13-00561-s001.zip › vetsci-4308689-supplementary.pdf]

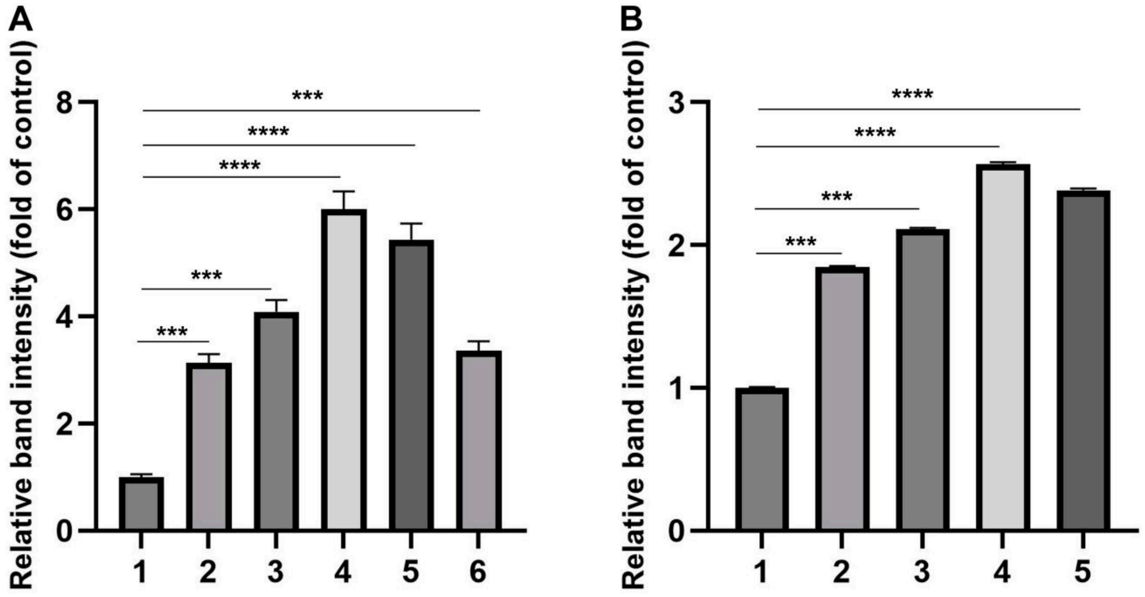

Supplementary Figure S1. Quantitative analysis of Basic-RPA amplification under different temperature and time conditions. (A) Densitometric analysis of Basic-RPA amplification products obtained under different reaction temperatures. Bars 1-6 represent 25, 30, 35, 37, 39, and 45 °C, respectively. (B) Densitometric analysis of Basic-RPA amplification products obtained at different reaction times. Bars 1-5 represent 10, 20, 25, 30, and 35 min, respectively. Relative band intensities were quantified by image analysis and normalized to group 1 in each panel. Data are shown as mean ± SEM. Statistical significance was determined by one-way ANOVA followed by a multiple-comparison test. Asterisks indicate statistically significant differences between the indicated groups (***, $P < 0.001$; ****, $P < 0.0001$).

Supplementary Table S1. Regional distribution and clinical classification of the 153 bovine milk samples included in the field validation study

Serial Number	Sample ID	Group	Culture	qPCR	RTF	LFD
1	HN1	Clinical	+	+	+	+
2	HN2	Clinical	-	-	-	-
3	HN3	Clinical	-	-	-	-
4	HN4	Clinical	+	+	+	+
5	HN5	Clinical	-	-	-	-
6	HN6	Clinical	-	-	-	-
7	HN7	Clinical	-	-	-	-
8	HN8	Clinical	-	-	-	-
9	HN9	Clinical	-	-	-	-
10	HN10	Clinical	-	-	-	-
11	HN11	Clinical	-	-	-	-
12	HN12	Clinical	-	-	-	-
13	HN13	Subclinical	-	-	-	-
14	HN14	Subclinical	+	+	+	+
15	HN15	Subclinical	-	-	-	-
16	HN16	Subclinical	-	-	-	-
17	HN17	Subclinical	+	+	+	+
18	HN18	Subclinical	-	-	-	-
19	HN19	Subclinical	-	+	+	+
20	LN1	Clinical	-	-	-	-
21	LN2	Clinical	-	+	+	+
22	LN3	Clinical	-	-	-	-

23	LN4	Clinical	-	-	-	-
24	LN5	Clinical	-	-	-	-
25	LN6	Clinical	+	+	+	+
26	LN7	Clinical	-	-	-	-
27	LN8	Clinical	-	-	-	-
28	LN9	Clinical	-	-	-	-
29	LN10	Subclinical	-	-	-	-
30	LN11	Subclinical	-	-	-	-
31	LN12	Subclinical	+	+	+	+
32	LN13	Subclinical	-	-	-	-
33	LN14	Subclinical	-	-	-	-
34	LN15	Subclinical	-	-	-	-
35	LN16	Subclinical	+	+	+	+
36	LN17	Subclinical	-	-	-	-
37	LN18	Subclinical	-	+	+	+
38	LN19	Subclinical	-	-	-	-
39	LN20	Subclinical	-	-	-	-
40	LN21	Subclinical	-	-	-	-
41	LN22	Health	-	-	-	-
42	LN23	Health	-	-	-	-
43	LN24	Health	-	-	-	-
44	LN25	Health	-	-	-	-
45	LN26	Health	-	-	-	-
46	LN27	Health	-	-	-	-
47	LN28	Health	-	-	-	-

48	LN29	Health	-	-	-	-
49	LN30	Health	-	-	-	-
50	LN31	Health	-	-	-	-
51	HLJ1	Clinical	-	-	-	-
52	HLJ2	Clinical	-	-	-	-
53	HLJ3	Clinical	+	+	+	+
54	HLJ4	Clinical	-	-	-	-
55	HLJ5	Clinical	-	-	-	-
56	HLJ6	Clinical	-	-	-	-
57	HLJ7	Clinical	+	+	+	+
58	HLJ8	Clinical	-	-	-	-
59	HLJ9	Clinical	-	-	-	-
60	HLJ10	Clinical	-	+	+	+
61	HLJ11	Clinical	-	-	-	-
62	HLJ12	Clinical	-	-	-	-
63	HLJ13	Clinical	-	-	-	-
64	HLJ14	Clinical	-	-	-	-
65	HLJ15	Clinical	+	+	+	+
66	HLJ16	Clinical	-	-	-	-
67	HLJ17	Clinical	-	-	-	-
68	HLJ18	Clinical	-	+	+	+
69	HLJ19	Clinical	-	-	-	-
70	HLJ20	Clinical	-	-	-	-
71	HLJ21	Clinical	-	-	-	-
72	HLJ22	Subclinical	-	-	-	-

73	HLJ23	Subclinical	-	-	-	-
74	HLJ24	Subclinical	+	+	+	+
75	HLJ25	Subclinical	-	-	-	-
76	HLJ26	Subclinical	-	-	-	-
77	HLJ27	Subclinical	+	+	+	+
78	HLJ28	Subclinical	-	-	-	-
79	HLJ29	Subclinical	-	-	-	-
80	HLJ30	Subclinical	+	+	+	+
81	HLJ31	Subclinical	-	+	+	+
82	HLJ32	Subclinical	-	-	-	-
83	HLJ33	Subclinical	-	-	-	-
84	HLJ34	Subclinical	-	-	-	-
85	HLJ35	Subclinical	-	+	+	+
86	HLJ36	Subclinical	-	-	-	-
87	HLJ37	Subclinical	-	-	-	-
88	HLJ38	Health	-	-	-	-
89	HLJ39	Health	-	-	-	-
90	HLJ40	Health	-	-	-	-
91	HLJ41	Health	-	-	-	-
92	HLJ42	Health	-	-	-	-
93	HLJ43	Health	-	-	-	-
94	HLJ44	Health	-	-	-	-
95	HLJ45	Health	-	-	-	-
96	SD1	Clinical	-	-	-	-
97	SD2	Clinical	-	-	-	-

98	SD3	Clinical	-	+	+	+
99	SD4	Clinical	-	-	-	-
100	SD5	Clinical	-	+	+	+
101	SD6	Clinical	-	-	-	-
102	SD7	Clinical	-	-	-	-
103	SD8	Clinical	+	+	+	+
104	SD9	Clinical	-	-	-	-
105	SD10	Clinical	-	-	-	-
106	SD11	Clinical	-	-	-	-
107	SD12	Clinical	+	+	+	+
108	SD13	Clinical	-	-	-	-
109	SD14	Clinical	-	-	-	-
110	SD15	Clinical	-	-	-	-
111	SD16	Subclinical	-	-	-	-
112	SD17	Subclinical	+	+	+	+
113	SD18	Subclinical	-	-	-	-
114	SD19	Subclinical	-	-	-	-
115	SD20	Subclinical	-	+	+	+
116	SD21	Subclinical	-	-	-	-
117	SD22	Subclinical	+	+	+	+
118	SD23	Subclinical	-	-	-	-
119	SD24	Subclinical	-	-	-	-

Supplementary Table S2 Comparison of RPA-PfAgo with other rapid molecular detection methods

Method	Main principle	Readout format	Equipment requirement	Main advantages	Main limitations
LAMP	Isothermal amplification using multiple primers	Colorimetric, turbidity, fluorescence, or LFD	Constant-temperature device	High amplification efficiency; suitable for rapid nucleic acid detection	Primer design is relatively complex; nonspecific amplification may occur; result interpretation can be affected by primer-dimer formation
RPA-LFD	Isothermal RPA amplification combined with lateral flow readout	Visual LFD	Low-temperature heating device; LFD strip	Rapid amplification; simple visual interpretation; relatively convenient for field screening	Specificity mainly depends on primer/probe design; nonspecific amplification may affect interpretation
CRISPR-Cas-based assay	Isothermal amplification combined with Cas-mediated programmable nucleic acid recognition	Fluorescence or LFD	Heating device; Cas enzyme system; fluorescence detector or LFD strip depending on format	High specificity; programmable target recognition; flexible readout options	Requires Cas enzymes and optimized reaction conditions; workflow may be relatively complex
RPA-PfAgo assay	RPA amplification followed by PfAgo-mediated programmable DNA cleavage	RTF or LFD	Low-temperature RPA step plus 95°C PfAgo reaction; fluorescence detector for RTF or LFD strip for visual readout	Additional target-recognition step after RPA; dual-readout flexibility; RTF provides higher analytical sensitivity and LFD enables visual interpretation	Two-step workflow; field use requires a compact heating device and DNA extraction
